# Neuroradiology training in EU: international survey of 31 countries within UEMS frame

**DOI:** 10.1186/s13244-020-00881-8

**Published:** 2020-05-22

**Authors:** Francesca B. Pizzini, Marek Sasiadek, Francesco Tanzi, Paolo Ricci

**Affiliations:** 1 UEMS Division of Neuroradiology, Radiology, Dept. of Diagnostic and Public Health, Verona University, Piazzale L.A. Scuro 10, 37134 Verona, Italy; 2 UEMS Division of Neuroradiology, Department of General and Interventional Radiology and Neuroradiology, Wroclaw Medical University, Wroclaw, Poland; 3 UEMS Section of Radiology, Brussels, Belgium; 4 UEMS Section of Radiology, Department of Radiology, Sapienza University of Rome, Rome, Italy

**Keywords:** Neuroradiology, Training, Survey

## Abstract

**Objective:**

To assess the current framework of interventional and diagnostic neuroradiology in Europe

**Methods:**

The UEMS (European Union of Medical Specialists) Section of Radiology and the subspecialty UEMS Division of Neuroradiology collected by e-mail a survey on the situation of diagnostic and Interventional Neuroradiology’ training and practice in Europe. The questionnaire was sent to the national delegates from 31 UEMS member countries, belonging to the European Union, the European Economic Area and the Council of Europe. In case of uncertain or discordant replies, the survey envisaged the involvement of neuroradiology scientific societies’ experts for providing a decisive answer.

**Results:**

A formal post-residency training in diagnostic and interventional neuroradiology is provided respectively by 12/31 and 20/31 of the European countries. Currently, for becoming neuroradiologist in a country without fellowship program, a radiologist should (1) get subspecialty credits, (2) follow training inside national or international neuroradiology departments, or (3) perform the main reporting activity in neuroradiology.

In nearly 2/3 of the States included in the survey, the neurointerventional procedures are provided by radiologists (22/31) and in the most frequent scenario a specific training in neurovascular is required to all radiologist or non-radiologist candidates (18/31).

**Conclusions:**

The European framework of neuroradiology’s training and practice that emerged through this survey is fragmented, but there is an increasing attention by European scientific societies and institutions to create a common path of training and practice that can guarantee high educational and patient care standards.

## Key points

- Neuroradiological (diagnostic and interventional) training in Europe is extremely heterogeneous.
- A survey of European situation is the base for a common action.
- A European neuroradiological training strategy can guarantee higher medical practice and assistance.

## Introduction

The European Union (EU) training system in diagnostic and interventional neuroradiology is heterogeneous and reflects the different national educational and training structures. European Union of Medical Specialists (UEMS), European Society of Neuroradiology-Diagnostic and Interventional (ESNR), and European Society of Minimally Invasive Neurological Therapy (ESMINT) have recently created a “European Charter for Interventional Neuroradiology” [1, 2], and during this last decade, scientific societies have updated the neuroradiology training skill, knowledge, competences, and attitudes within the European Training curriculum [3] and have been increasing the coordination of training courses and are implementing standardized exams in neuroradiology [4].

Despite this positive trend, in EU, there is still no official supervising institution, as the Accreditation Council for Graduate Medical Education (ACGME) in the United States of America (USA), which sets common standards of training and periodically reviews them. While in Europe the path of an aspiring neuroradiologist is not standardized, in the USA, after the completion of an ACGME-accredited radiology residency program, a candidate can start an additional training for subspecialty expertise [5]. Fellowships are offered in the USA in both diagnostic and interventional neuroradiology; the first one is ACMGE regulated, and it is a prerequisite (for 1 year) for the latter. To date, only few neurointerventional programs in the USA have pursued the ACGME accreditation pathway, for different reasons [5]. But an alternative accreditation and certification program have been proposed under the aegis of the Committee for Advanced Subspecialty Training (CAST) of the Society of Neurological Surgeons (SNS) [5].

Since there is currently increasing attention to guarantee a widespread and high-level training and practice across all countries, we launched this survey under the UEMS umbrella to investigate the European situation, intending to outline and summarize the variegated reality and hoping that this will lead to a synergistic educational and clinical practice response.

## Materials and methods

The survey was realized within the European Union of Medical Specialists (UEMS) Radiology Section and Neuroradiology Division, and it was focused on the situation of diagnostic and interventional neuroradiology training and practice in Europe, in view of the approval of the European Training Requirements in Interventional Neuroradiology (April 13, 2019) [1, 2].

**Fig. 1:**
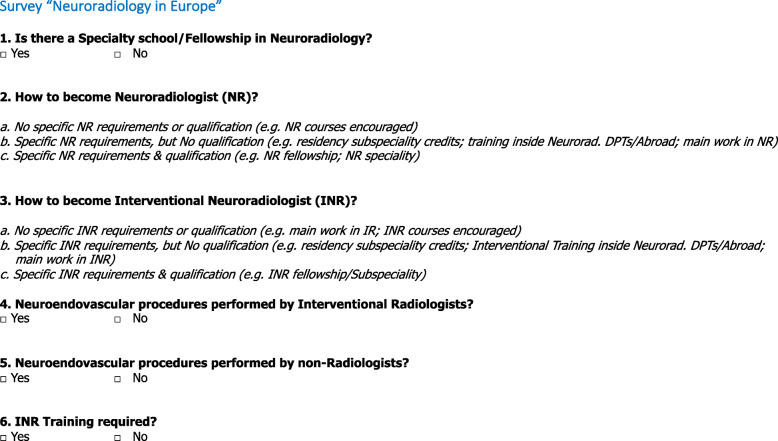
Template of the survey

It was launched in September 2018, and it targeted the national delegates from 31 UEMS member countries—from 1 to 3 responders for each country—and in case of multiple and discordant replies, it was also sent to scientific societies referents provided by European or national neuroradiology societies for a final decision (i.e., France, Italy, Switzerland and UK, asterisked in Table 1). The questionnaire highlighted how significant differences in neuroradiology and interventional training—this latter in endovascular procedures as treatment of aneurysms, acute ischemic stroke, extracranial and intracranial angioplasty/stenting, and embolization of arteriovenous malformation and fistulas and of external carotid (as listed in [1, 2])—and practice still persist in Europe. As for general categorization of responses, the format (Fig. 1) was a two-choice (yes or no for questions number 1, 4, 5, and 6) or a multiple-choice (for questions number 2 and 3) questionnaire which covered and synthetized the main possible scenarios.

**Table 1:** Survey results

Country	No. of respondents	Specialty school/fellowship in neuroradiology	How to become neuroradiologist	How to become interventional neuroradiologist	Neuroendovascular procedures performed by interventional radiologists	Neuroendovascular procedures performed by non-radiologists	INR training required
Austria	3	No	No specific NR requirements or qualification.	No specific INR requirements or qualification. Certification of neuro-interventionalists and training sites based on existing modular certification concept by the scientific societies ÖGIR (Austrian society for interventional radiology) and ÖGNR (Austrian society for neuroradiology) is highly recommended.	Yes	Yes	No
Belgium	2	No	No specific NR requirements or qualification.	No specific INR requirements or qualification.	Yes	Yes	No
Bulgaria	2	No	No specific NR requirements or qualification.	Specific INR requirements, but No qualification.	Yes	Yes	No, but it is in progress.
Croatia	1	Yes	Specific NR requirements & qualification.	No specific INR requirements or qualification.	Yes	No	Yes, but no formal
Cyprus	1	Yes	Specific NR requirements, but No qualification.	Specific INR requirements, but No qualification.	Yes	No	Yes
Czech Republic	1	No, it used to be in the past.	No specific NR requirements or qualification.	No specific INR requirements or qualification. INR is part of Interventional Radiology training.	Yes	No	No
Denmark	1	No	No specific NR requirements or qualification.	Specific INR requirements, but No qualification.	Yes	No	Yes: No formal fellowship is available, but training is required before independent work as INR specialist is performed.
Estonia	1	No	No specific NR requirements or qualification.	Specific INR requirements, but No qualification.	Yes	Yes	Yes
Finland	3	Yes	Specific NR requirements & qualification.	Specific INR requirements, but No qualification.	Yes	No	Yes
France*	3	No	Specific NR requirements, but No qualification.	Specific INR requirements, but No qualification.	Yes	Yes	Yes
Germany	3	Yes	Specific NR requirements & qualification.	Specific INR requirements or qualification.	Yes	No	Yes
Greece	2	No. But in the process of the subspecialty of diagnostic and interventional neuroradiology to be approved.	No specific NR requirements or qualification.	Specific INR requirements & qualification. The qualification is currently obtained by training in other countries that provide INR qualifications and is approved by the Ministry of Health.	No. There is a discussion that Interventional Radiologists get involved in stroke after a minimum of 9-month training.	Yes	Yes
Hungary	1	Yes	Specific NR requirements & qualification. It is a secondary board examination following board exam in Radiology.	Specific INR requirements & qualification. See point 6.	Yes, after completion of the special competency license training and examination in Neurointerventions.	Yes, after completion of the special competency license training and examination in Neurointerventions.	Yes, it is a special competency license, requiring a 2 years fulltime clinical training in INR following a board exam in either radiology, neuroradiology, neurology, neurosurgery or cardiology.
Iceland	1	No	Specific NR requirements & qualification.	Specific INR requirements & qualification.	No	No	No
Ireland	1	Yes: no specialty school exists, but fellowship training in Neuroradiology is provided.	Specific NR requirements & qualification.	Specific INR requirements, but No qualification. Must have completed 2 years of fellowship training in neuroradiology.	No	No	Yes
Italy*	4	No	Specific NR requirements, but No qualification.	Specific INR requirements, but No qualification.	Yes	Yes	No
Latvia	1	Yes	Specific NR requirements & qualification.	Specific INR requirements, but No qualification.	Yes	No	Yes
Lithuania	1	No	No specific NR requirements or qualification.	No specific INR requirements or qualification. INR procedures could be performed by any interventional radiologist.	Yes	Yes. In smaller centres, cardiologists perform thrombectomy (only) in case of stroke.	No
Luxembourg	1	No	Specific NR requirements, but No qualification.	Specific INR requirements, but No qualification.	Yes	Yes	No
Malta	1	No	Specific NR requirements, but No qualification.	Specific INR requirements, but No qualification.	No	No	Yes
Netherlands	1	Yes	Specific NR requirements & qualification.	Specific INR requirements & qualification.	No, except for endovascular treatment (stroke).	Yes	Yes (dedicated fellowship for INR, which can be entered by certified Radiologists, Neurologists, and Neurosurgeons).
Norway	1	No	No specific NR requirements or qualification.	No specific INR requirements or qualification.	Yes	Yes	No
Poland	3	No	No specific NR requirements or qualification.	Specific INR requirements & qualification (certificate provided by Section of Interventional Neuroradiology, which is a Subdivision of Polish Medical Society of Radiology).	Yes, only stroke.	Yes	Yes
Portugal	1	Yes	Specific NR requirements & qualification. Neuroradiology is an independent speciality with a five-year training programme and a final examination by a national jury.	Specific INR requirements & qualification.	No. Only Neuroradiologists can perform neuro intervention.	No. Only Neuroradiologists can perform these interventions as answered previously.	Yes
Romania	1	No	Specific NR requirements, but No qualification. From 2018 NR is part of specialization of Interventional Radiology.	Specific INR requirements & qualification. Mandatory training in NR and INR, and candidates must pass an exam in order to perform Neurointerventional procedures.	Yes	Yes, very few cases (neurosurgeons).	Yes, but probably no more in the future.
Slovenia	1	No	Specific NR requirements, but No qualification.	Specific INR requirements, but No qualification.	Yes	No	Yes
Spain	2	No	No specific NR requirements or qualification.	Specific INR requirements, but No qualification.	Yes	Yes	No. However, most Neuro-interventionalists have received training from Interventio-nal Neuroradiologists in Academic/University Hospitals.
Sweden	2	Yes	Specific NR requirements & qualification.	Specific INR requirements, but No qualification.	No	Yes	Yes
Switzerland*	3	Yes	Specific NR requirements & qualification & examination.	Specific INR requirements & qualification & examination.	No (unless in a certified neurovascular centre under the supervision of a certified neuroradiologist).	No (unless in a certified neurovascular centre under the supervision of a certified neuroradiologist).	Yes
Turkey	1	No	Specific NR requirements & qualification.	Specific INR requirements & qualification.	Yes	Yes	No
UK*	4	Yes: no specialty school exists, but fellowship training in Neuroradiology is provided.	Specific NR requirements, but No qualification.	Specific INR requirements, but No qualification.	Not yet. (currently there are body interventional radiologists training for thrombectomy).	No	Yes

*Countries where the involvement of additional referents from European or National scientific societies was necessary due to the uncertainty or discordance of the UEMS delegate’s answer/s

Regarding the 2 open questions (2. “How to become Neuroradiologist?” and 3. “How to become Interventional Neuroradiologist?”), these following three different scenarios have been envisaged and proposed:

1. *No specific Neuroradiology (NR)/Interventional Neuroradiology (INR) requirements or qualification*: this answer indicates that there is not a NR/INR fellowship or specialty or program, but the participation to NR/INR courses is encouraged for being considered NR or INR.
2. *Specific NR/INR requirements, no qualification*, when subspecialty credits or training inside neuroradiology departments (national or international) or a main neuroradiology reporting activity are required, but there is not a specific fellowship or specialistic degrees required.
3. *Specific NR/INR requirements and qualification*, when a recognized fellowship or specialistic degree and training program are required.

The survey has been circulating by e-mail among respondents for 11 months after the launch, and it has been further presented and discussed in the UEMS Section and Division meetings held in March (Vienna) and September (Rome) 2019, then completed and finalized after this latter one.

## Results

**Fig. 2:**
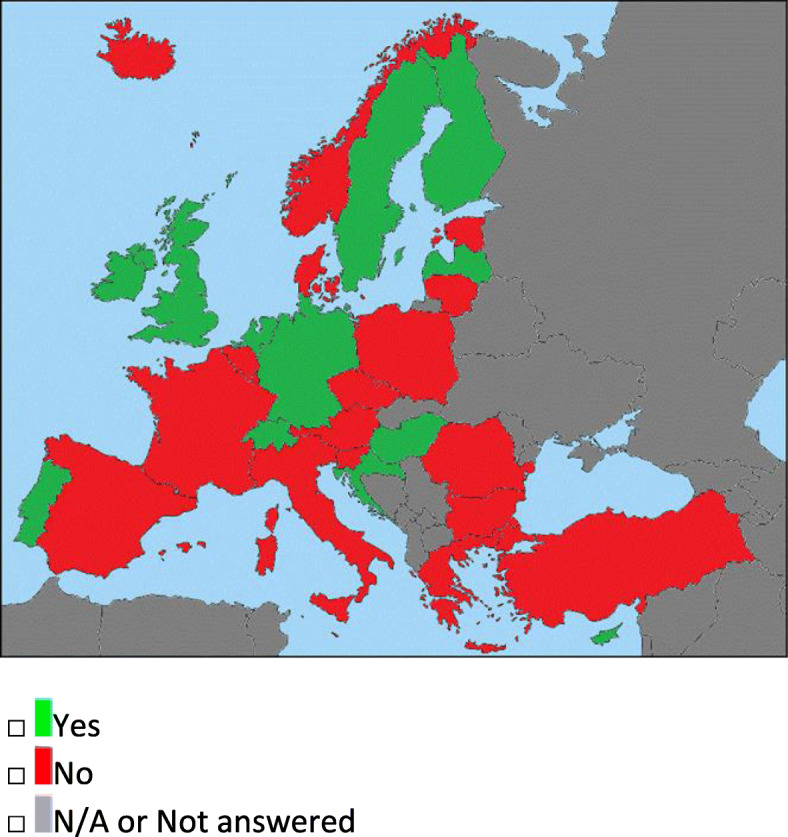
Question 1: Specialty school/fellowship/training in neuroradiology. In red, the countries where a specialty training does not exist, while in green the ones where it is available. In gray, the countries which have not answered or which were not included in the survey from the beginning

**Fig. 3:**
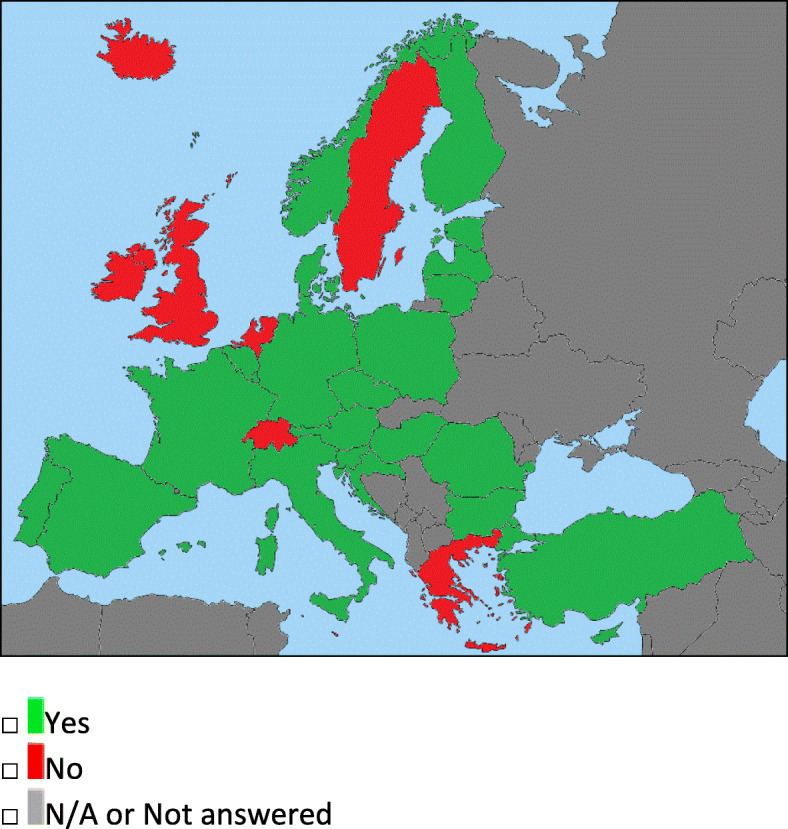
Question 4: Neuroendovascular procedures performed by interventional radiologists. In green the countries where the interventional procedures are performed by interventional radiologists (in Portugal only by neuroradiologist), while in red, the ones where the procedures are performed by non-radiologist. In gray, the countries which have not answered or which were not included in the survey from the beginning

**Fig. 4:**
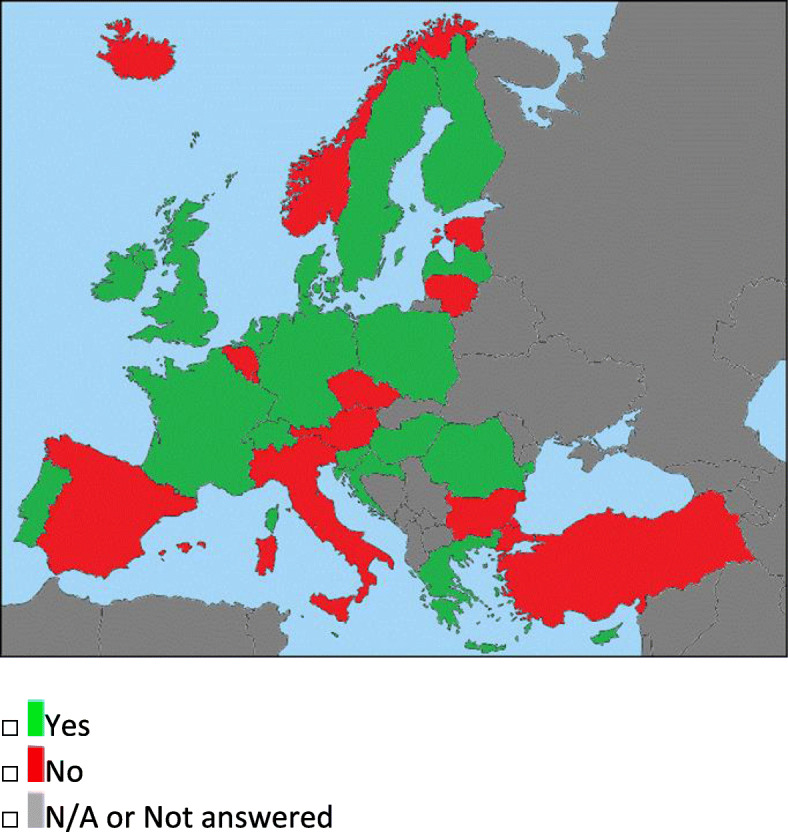
Question 6: INR training required. In green, the countries where a specific interventional training is required, while in red, the ones where it is not. In gray, the countries which have not answered or which were not included in the survey from the beginning

The total number of the UEMS countries involved was 31, and each country provided a unique answer. The survey was sent to 53 UEMS delegates, but, in case of uncertain answer or of discordant answers, it was also forwarded to the scientific societies’ experts. The results of the inquiry are summarized in Table 1 and in the main conceptual maps (Figs. 2, 3, and 4), and they are also visible on the UEMS neuroradiology division website: https://neuro.uemsradiology.eu/specialty-in-europe/#education-and-training

### Neuroradiology training (questions 1 and 2)

A neuroradiology specialty school, a fellowship, or a specific training program is required in 12 of 31 countries (Fig. 2).

In 10 nations, a school or fellowship is provided, while in the UK and Ireland there is a specific neuroradiology training to follow. Greece will be probably one of the next countries to get a specific NR training, since it is in the process of approving the subspecialty of diagnostic and interventional neuroradiology.

In countries where there is not a fellowship/school or training (Table 1), the NR expertise could be reached through:

1. Residency subspeciality credits or training inside national or international recognized neuroradiology training centers or if the main work is in neuroradiology (*specific NR requirements, no qualification answer*)
2. Participation to neuroradiology courses (*no specific NR requirements or qualification answer*)

### Interventional neuroradiology training and practice (questions 3–6)

The most frequent scenario (Fig. 3) is the one in which the *Interventional Neuroradiology* procedures are covered by interventional radiologists (22), with the specificity of Portugal—where only neuroradiologists can perform neurointervention—and of some nations—where there are further requirements (e.g., a specific training in neurointerventions or under NR supervision, as in Hungary and Switzerland) or where the procedures are limited to stroke treatment (i.e., Poland). In 17/31 countries (often only in few centers), neurovascular stroke procedures are performed also by non-radiologist—mainly neurosurgeons or cardiologists (Table 1).

Twenty nations require a specific INR training (Fig. 4; Table 1) before independent work as INR specialist could be performed (i.e., Denmark). This could be also a qualification such as a dedicated fellowship for interventional neuroradiology, which can be entered by certified radiologists, neurologists, and neurosurgeons (The Netherlands), or a special competency license (Hungary).

In all the other countries where the qualification is not required, the INR training could be achieved in a similar way to the NR (i.e., (1) through subspeciality credits or training inside national or international recognized INR Training centers or if the main workload is in INR*—specific INR requirements, no qualification answer—*or (2) participation to INR courses*—No specific NR requirements or qualification answer*-).

## Discussion

This is the first survey to explore the European interventional and diagnostic training in different countries, and the questionnaire development and structure tried to synthesize and cover the main differences—but also to cluster some similarities—between neuroradiology educational programs and practices.

There is a wide variety of training in neuroradiology offering all over the world due to a widely variation of the structure and regulation of the training and, though less, to the variation of its duration [5]. And throughout the world, the main differences of training programs are not limited to neuroradiology, but also to the structure and duration of the medical school curricula and radiology residency programs (teaching modules, duration, a faculty-to-resident ratio) [5].

Regarding the specific training in neuroradiology in Europe, some nations have a specialized fellowship only in interventional or in both diagnostic and interventional (Table 1) with the specificity of Portugal where neuroradiologists follow a separate training track and their residency is separate from the general radiology.

The definition of standards of training and practice can guarantee highest quality medical care and safety. This can be reached by accreditation and certification of training programs. There is a worldwide interest in the production of consensus documents in neurointerventional (NI) training which see the USA as the leader. Different neurosurgical, neuroradiological, and neurologic societies connected to NI have agreed on a training curriculum and related documents [6], proposing individual training, certification and requirements, and resources.

In the USA, there are also structured diagnostic radiology educational programs and neuroradiology fellowship training which are ACGMR-approved and based on concrete guidelines [7–9], including the implementation of national certifying examinations [7].

The survey’s results evidenced Europe’s lack of guidelines and standardization of training and practice, even if training and educational courses are provided in NI by ESMINT (ECMINT, European Course in Minimally Invasive Neurological Therapy; EXMINT, European Stroke Course; Stroke Winter School and ESMINT fellowship) [10] and ESNR, the latter organizing also diagnostic courses for different levels of learners (i.e., European School of Radiology (ESOR), European Course in Neuroradiology (ECNR), Higher qualifications) [4]. The first-level (ESOR) course can be addressed to residents, and it is organized on a yearly basis as a 2-day course, while ECNR is recommended to radiologist with a minimum of neuroradiology training and divided in different modules of learning (1st, embryology/anatomy/malformations/genetics; 2nd, tumors and tumor-like lesions; 3rd, vascular diseases; 4th, trauma/degenerative/metabolic/inflammatory), while third-level courses are designed for neuroradiologists and provide higher qualification in interventional or spine, pediatric, or head and neck neuroradiology [4].

### Generalizability of results

The limitations of this survey are based on the bias for the responders that were selected within the UEMS and main European societies’ representatives. In addition, some answers may not summarize all the complexity of one national reality, and sometimes, there were some differences in the replies from the referents coming from the same country. Anyway, in case of inconsistent answers, we were able to cross-check the validity of these replies by interviewing national representatives of neuroradiology societies.

In conclusion*,* this is the first study providing a picture of the current scenario of the neuroradiology training and practice in Europe. Active intervention is needed for harmonizing the heterogeneity of organization of NR training between the EU countries and for creating a common standard of knowledge. That can be achieved through the implementation of a standardized European program and a Charter for educational and training and then through establishment of a common certification and license.

## Data Availability

The datasets used and/or analyzed during the current study are available online https://neuro.uemsradiology.eu/specialty-in-europe/#education-and-training

## Abbreviations

ACGME: Accreditation Council for Graduate Medical Education
CAST: Committee for Advanced Subspecialty Training
EDiNR: European Diploma in Neuroradiology
ESMINT: European Society of Minimally Invasive Neurological Therapy
ESNR: European Society of Neuroradiology-Diagnostic and Interventional
ESOR: European School of Radiology
EU: European Union
INR: Interventional neuroradiology
NI: Neurointerventional
NR: Neuroradiology
SNS: Society of Neurological Surgeons
UEMS: Union of Medical Specialists
USA: United States of America
